# Ten simple rules on how to create open access and reproducible molecular simulations of biological systems

**DOI:** 10.1371/journal.pcbi.1006649

**Published:** 2019-01-17

**Authors:** Arne Elofsson, Berk Hess, Erik Lindahl, Alexey Onufriev, David van der Spoel, Anders Wallqvist

**Affiliations:** 1 Department of Biochemistry and Biophysics, Science for life Laboratory, Stockholm University, Solna, Sweden; 2 Department of Physics, Swedish e-Science Research Center, KTH Royal Institute of Technology, Stockholm, Sweden; 3 Departments of Computer Science and Physics, Center for Soft Matter and Biological Physics, Virginia Tech, Blacksburg, Virginia, United States of America; 4 Uppsala Centre for Computational Chemistry, Science for Life Laboratory, Department of Cell and Molecular Biology, Uppsala University, Uppsala, Sweden; 5 Department of Defense Biotechnology High Performance Computing Software Applications Institute, Telemedicine and Advanced Technology Research Center, U.S. Army Medical Research and Materiel Command, Fort Detrick, Maryland, United States of America; Dassault Systemes BIOVIA, UNITED STATES

All *PLOS* journals have an open data policy that, amongst other things, states that all data and related metadata underlying the findings reported in a submitted manuscript should be deposited in an appropriate public repository, or for smaller datasets, as supporting information. This should obviously apply to computational methods as well, but unfortunately this is not always applied in practice, although it is of greatest importance for the scientific quality of simulations [1] and other modeling projects [2].

Molecular dynamics [3] and other type of simulations [2,4] have become a fundamental part of life sciences. The simulations are dependent on a number of parameters such as force fields, initial configurations, simulation protocols, and software. Researchers have different opinions about the types of software they prefer, and in general, we believe authors should be free to choose the tools that best fit their needs. However, as scientists, we also have a common obligation to critically test each other’s statements to find mistakes (including errors in the algorithms and bugs in the code), which can be exemplified by a heated debate over simulations of supercooled water that ended up being due to a subtle algorithmic issue [5], and we believe *PLOS* has a particularly strong responsibility to lead this development even if it might cause some short-term grief [6].

In particular, all published results should, in principle, be possible to reproduce independently by scientists in other labs using different tools. To ensure this, we propose a set of standards that any publication in *PLOS Computational Biology*, and hopefully, publications in other journals as well, should follow. We do believe that the sooner such policies are widely adapted, the more open and collaborative science will flourish [7].

These 10 simple rules should not be limited to molecular dynamics but also include Monte Carlo simulations, quantum mechanics calculations, molecular docking, and any other computational methods involving computations on biological molecules.

## Rule 1: The simulation protocol should be provided

The complete set of input files that are used in the simulations should be provided, either as supplementary material or preferably through a publicly available repository.

## Rule 2: Topology and parameters should be accessible for everyone

All topology and parameter files used in the simulations should be provided and made publicly available so they can be implemented and tested with a different program if necessary. That means the files should either be in human-readable format or the conversion rules should be be publically available.

## Rule 3: Initial coordinate files should be included

All simulations are strongly dependent on the initial conditions [8]. To ensure maximum reproducibility, the authors should provide the input coordinate files for the simulations in the appropriate formats for the software used. The input files to initiate the simulations should be provided in a format ensuring that a reader can repeat all parts of the calculation workflow himself or herself. That means the files should either be in human-readable format or the conversion rules should be publicly available.

## Rule 4: Full information about all software used needs to be provided

Reviewers and readers must be able to reproduce results with as much detail as possible. This means authors need to provide enough details so that the work can be repeated with widely available programs or that the software is provided. In particular, indicate the specific version of the software package used in the simulation. To further improve reproducibility, we encourage software authors to add information about compilers, flags, and the hardware used to log files.

## Rule 5: Simulation results should be deposited in a database

Following the *PLOS* editorial policy for data access, the authors should deposit representative snapshots from the trajectories and/or simulations in findable, accessible, interoperable, reusable (FAIR) public repositories. The deposited snapshots must be dense enough so that the reported biological insights are supported with the same statistical error margin as originally reported and so that new analyses and publications can be performed using the deposited data.

## Rule 6: Results should be easy to reproduce

Although the advantages of open source software are plentiful [9], many authors still use commercial software for simulations. However, in these cases, if the software used is not publicly available, the simulation method must be provided or already published in sufficient details so that the results can be reproduced within reported margin of error using publicly available software. Software and scripts used for analysis must also be made publicly available.

## Rule 7: In docking studies, details should be included

For all studies including screening and docking, the complete set of molecules tested as well as the scoring functions used and the high-ranking poses should be publicly available either as databases or detailed descriptions.

## Rule 8: In quantum mechanics calculations, all energies should be included in the results

For quantum mechanics studies, the authors need to provide the following information: absolute energies and energy breakdowns, the level of theory used, the basis set used, the optimization algorithm used, and coordinates of all optimized stationary points. Ideally, the archive entry for each calculation will be provided alongside the coordinates.

## Rule 9: All sampling-based results should be evaluated using proper statistics

As in all scientific studies, statistical rigor is necessary in computational studies to evaluate the significance of an observation—in particular, for any method based on sampling, such as molecular dynamics or Monte Carlo simulations. Appropriate estimates of statistical uncertainty are therefore necessary and should be included for each relevant finding.

## Rule 10: Be Nice

Remember that we all are a community, so sharing your data, methodologies, software, and results in such a way so that others can use it will make the entire community thrive. This also applies to the readers—they should not expect unlimited support for using in-house software or methods. Just the fact that it is available provides an important resource for the community.

## Conclusion

As a result of these discussions, *PLOS Computational Biology* has made the following extension to the *PLOS* data sharing policies:

- The authors should provide a README file with a list of included files and/or links to publicly available repositories along with their brief description.
- The authors should describe all software used including the specific version(s) used in the work and how it can be obtained.
- *PLOS* expects researchers to share software and scripts needed for the work. If this cannot be made publicly available (e.g., due to licenses), the simulation method should be provided in sufficient detail so the results can, in principle, be reproduced using publicly available software.
- The authors should provide the complete set of input files used to initiate the calculations, including input coordinates, topologies, and parameter files. These files must be provided in human-readable formats and should preferably be included as supplementary material.
- The authors should deposit trajectories in a public repository according to FAIR data principles [10]. Examples of such databases include ModEL [11], Nomad (https://nomad-repository.eu/), and the Dryad repository (https://datadryad.org/).
